# Supplementary material for: Unraveling the Mechanisms of Sensitivity to Anti-FGF Therapies in Imatinib-Resistant Gastrointestinal Stromal Tumors (GIST) Lacking Secondary KIT Mutations
Source: Cancers (Basel). 2023 Nov 9;15(22):5354. doi: 10.3390/cancers15225354 (PMC10670741; doi:10.3390/cancers15225354)
Supplement: Supplementary file 1 [file cancers-15-05354-s001.zip › File S1.pdf]

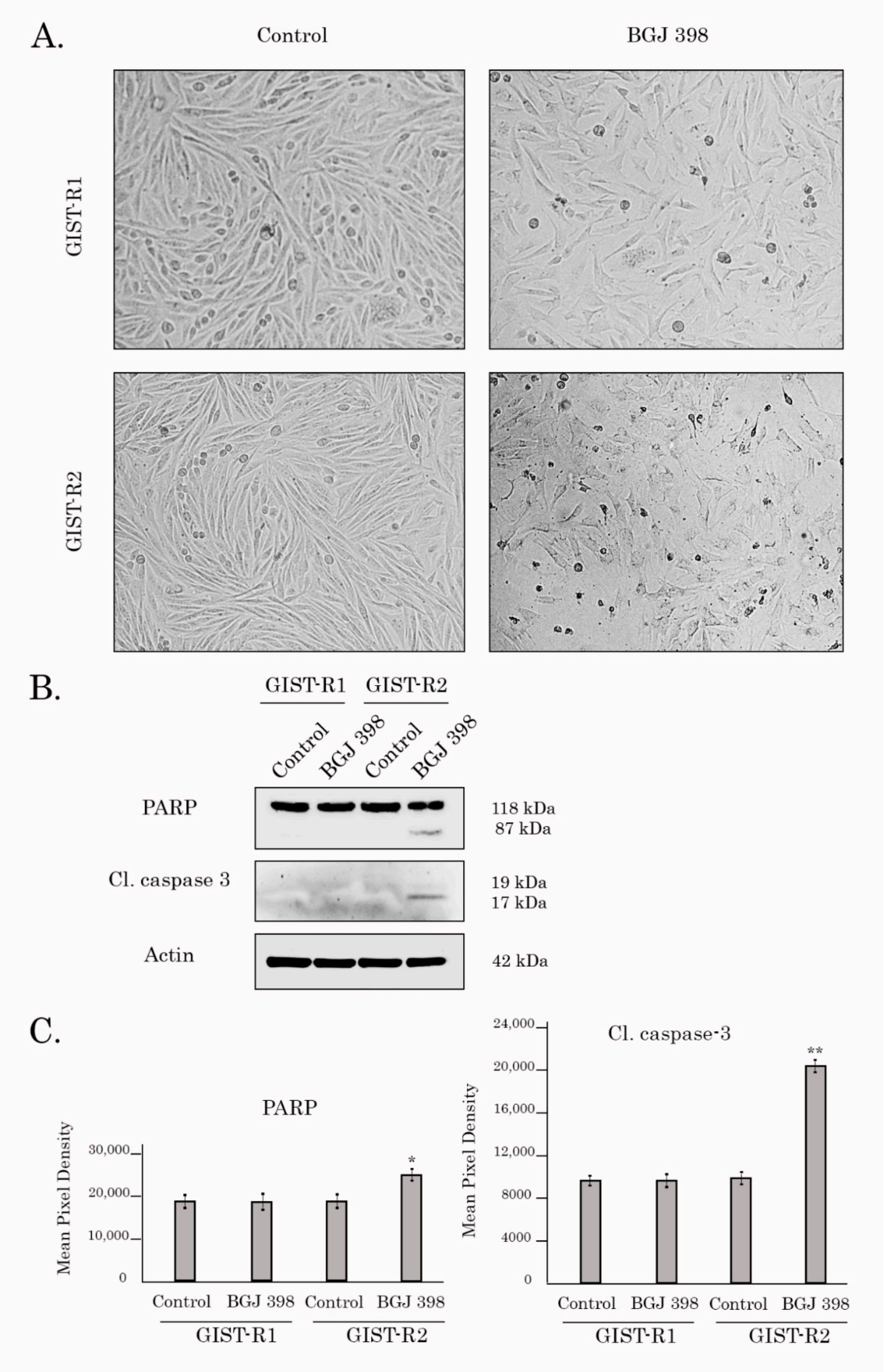

Supplementary Figure S1. Changes in IM-resistant GIST cells R-1 and R-2. **(A)** GIST-R1 and GIST-R2 cells were treated with solvent (DMSO) (control), BGJ 398 (1 μM) for 48 h and subjected to the light microscopy (Leica, 10×). **(B)** Study of apoptosis markers (cleaved forms of PARP and caspase-3) in GIST R-1 and R-2 cells using Western blotting. Cells were cultured in the presence of BGJ398 (1 μM) for 48 h. **(C)** Quantification by mean pixel density of cleaved forms of PARP and caspase-3 in GIST-R1 and R2 cells. Values are means ± SD, N = 3. *: $p < 0.05$, **: $p < 0.01$ vs untreated cells.

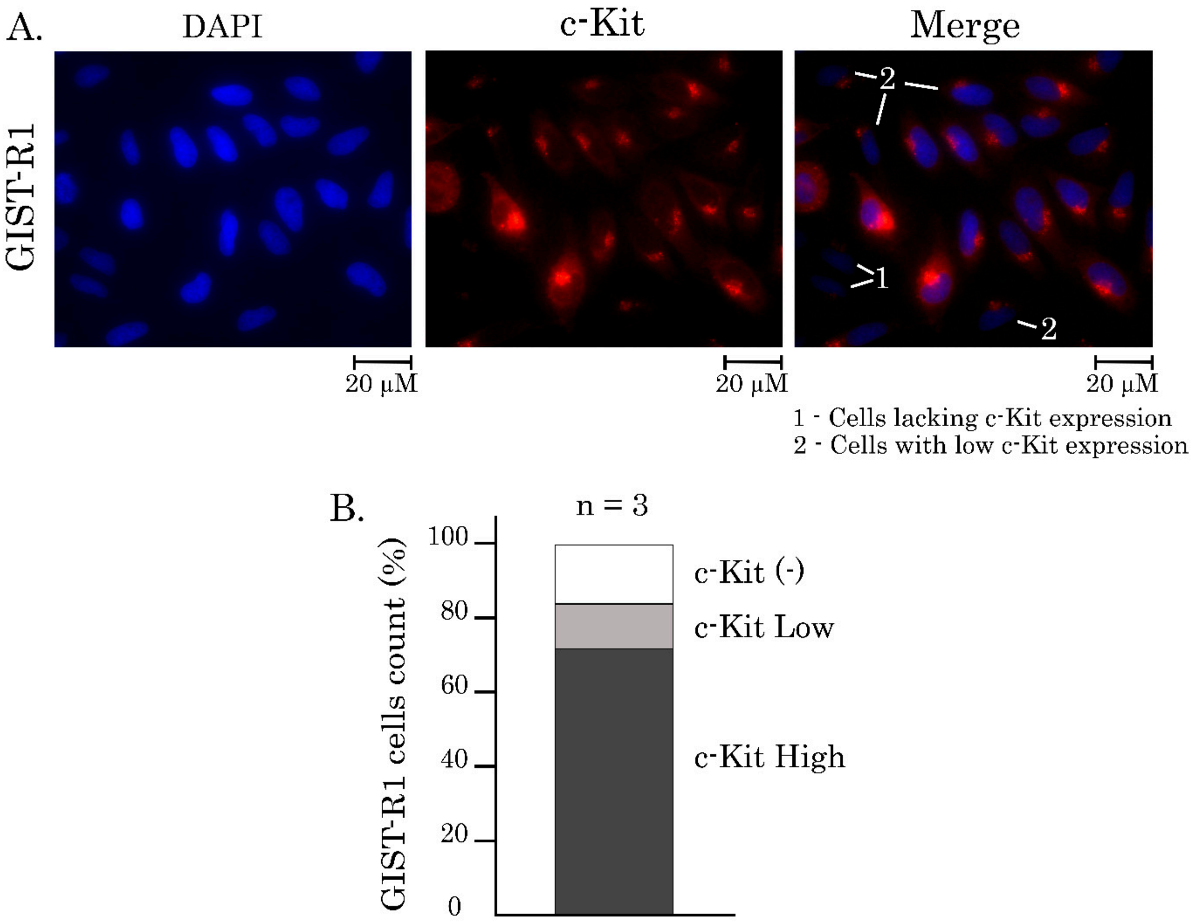

Supplementary Figure S2. Study of c-Kit expression in GIST-R1 cells. **(A)** Immunofluorescence staining of c-Kit expression in GIST-R1 cells. Cell nuclei were outlined by staining with DAPI. Magnification 40×, scale bars 20 μM. **(B)** The graph below demonstrates a comparative analysis of c-Kit expression in GIST-R1 cells (n = 3).

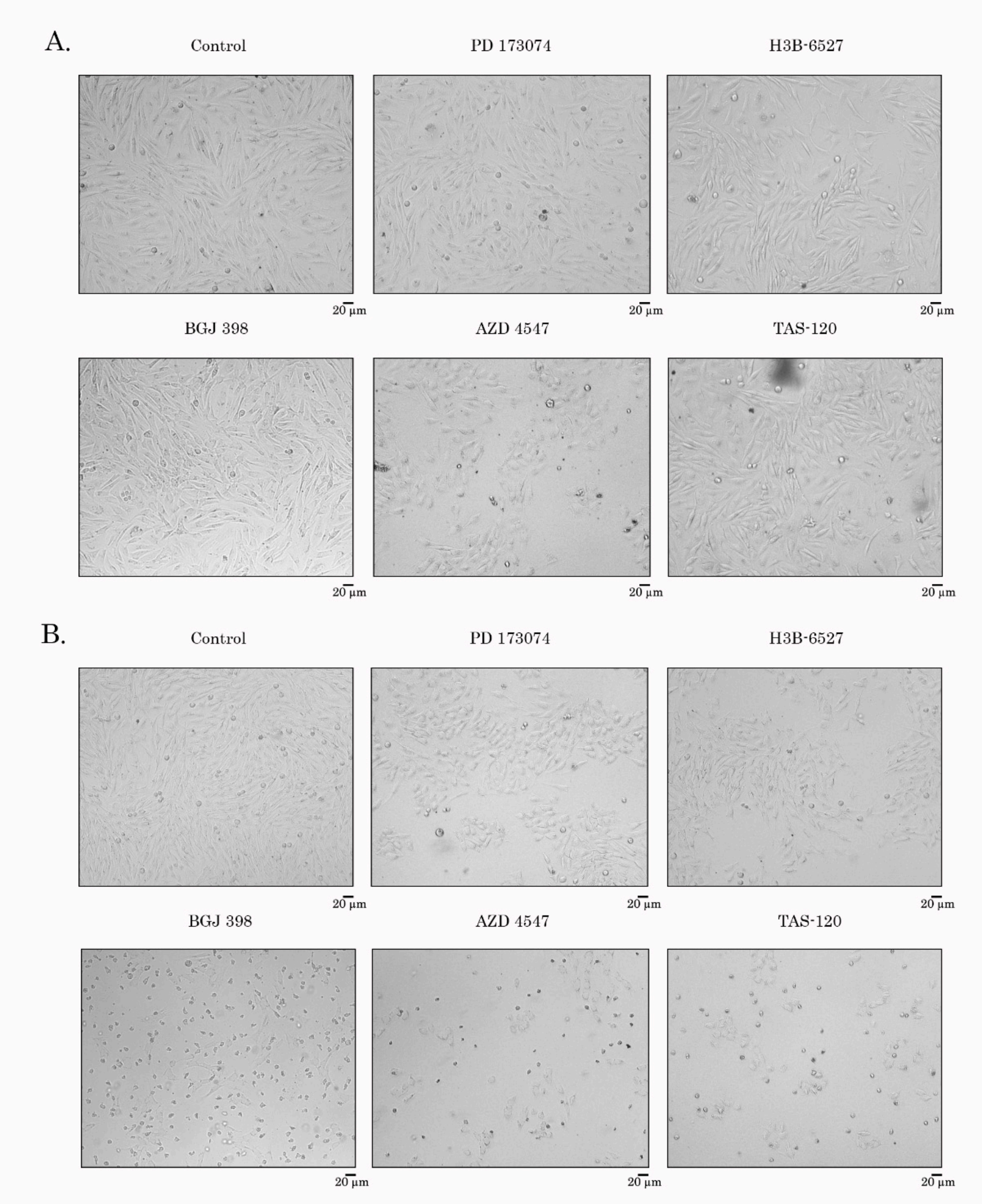

Supplementary Figure S3. Morphological changes in IM-resistant GIST cells treated with FGFRi. GIST-R1 **(A)** and GIST-R2 **(B)** cells were treated with solvent (DMSO) (control), PD 173074 (10 μM), H3B-6527 (1 μM), BGJ 398 (1 μM), AZD 4547 (1 μM) and TAS-120 (1 μM) for 48 h and subjected to the light microscopy.

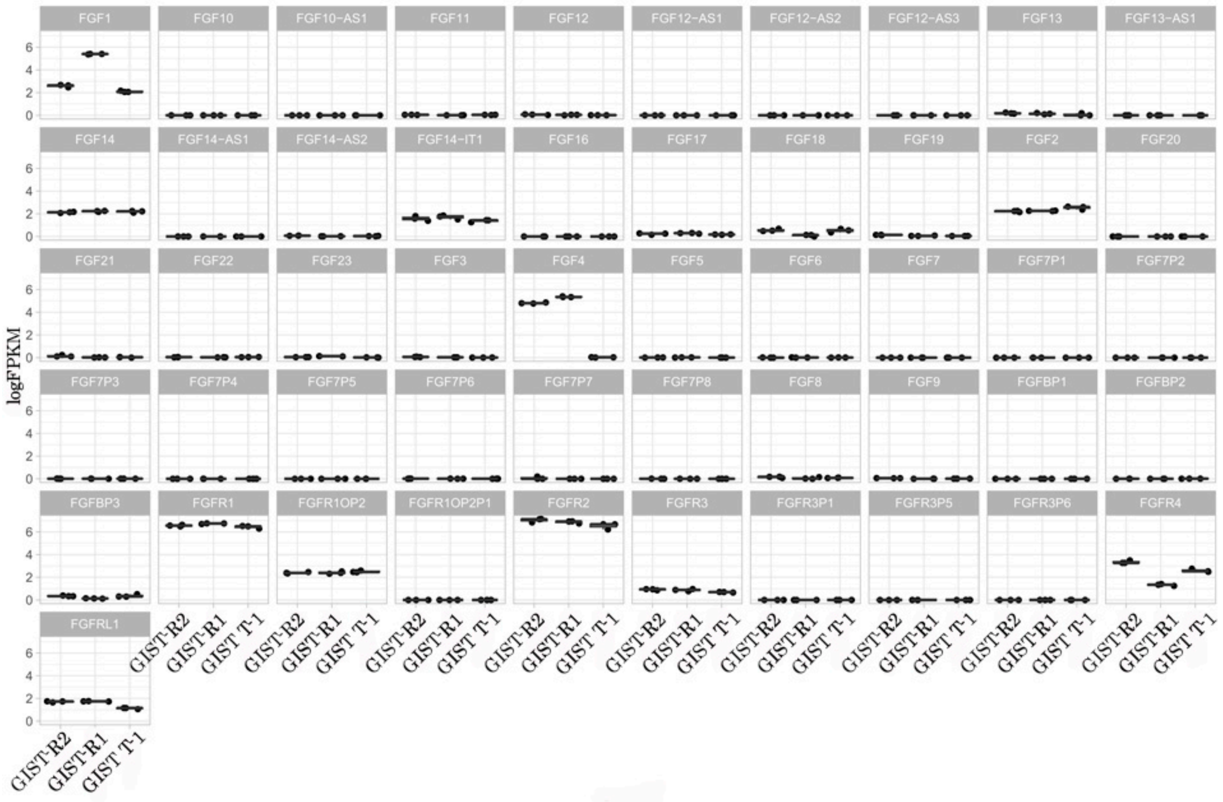

Supplementary Figure S4. Expression profiles of *FGF* and *FGFR* genes.

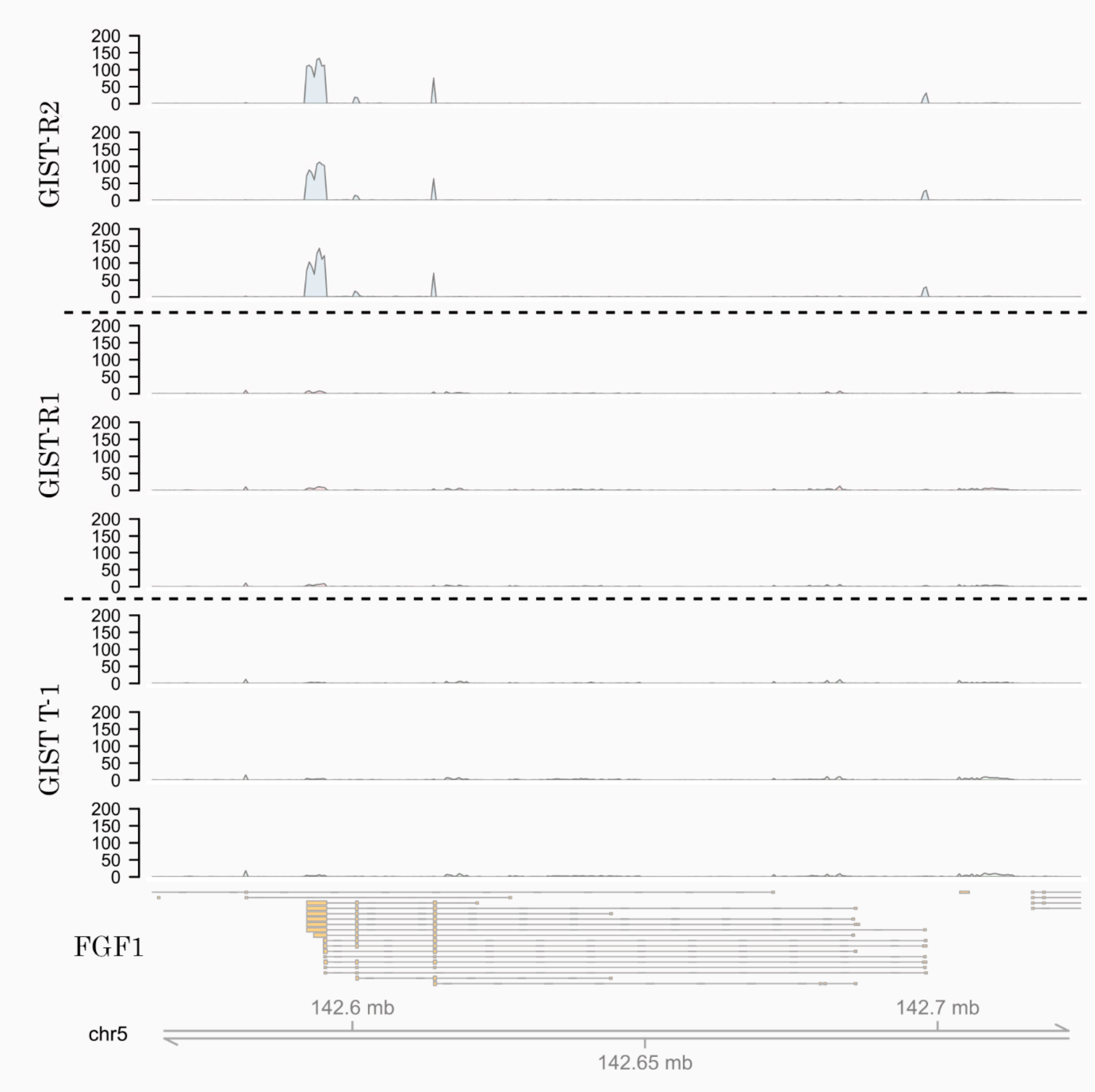

Supplementary Figure S5. Genomic view on *FGF1* gene showing its coverage in GIST cell lines.

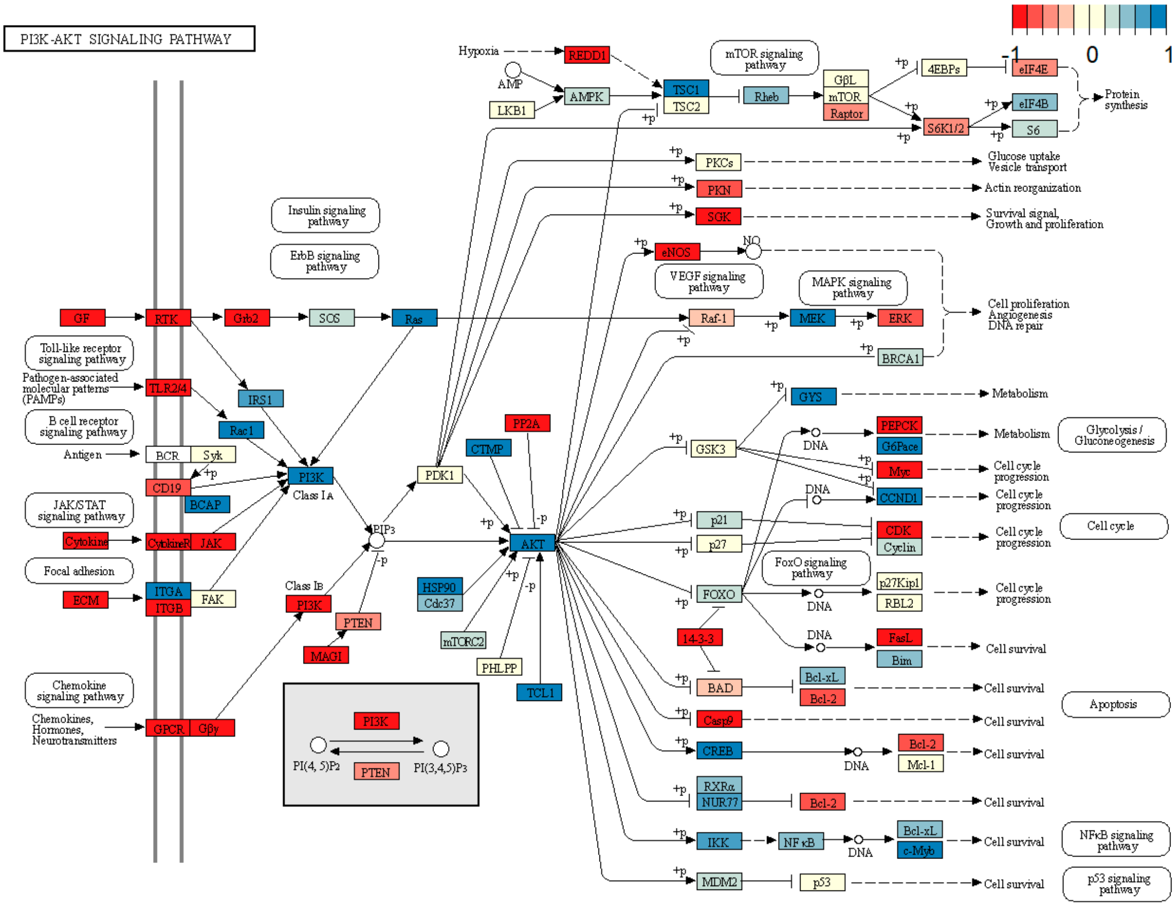

Supplementary Figure S6. PI3K-AKT signaling pathway changes between GIST-R1 cells (red, scaled expression < 0) and GIST-R2 cells (blue, scaled expression > 0).

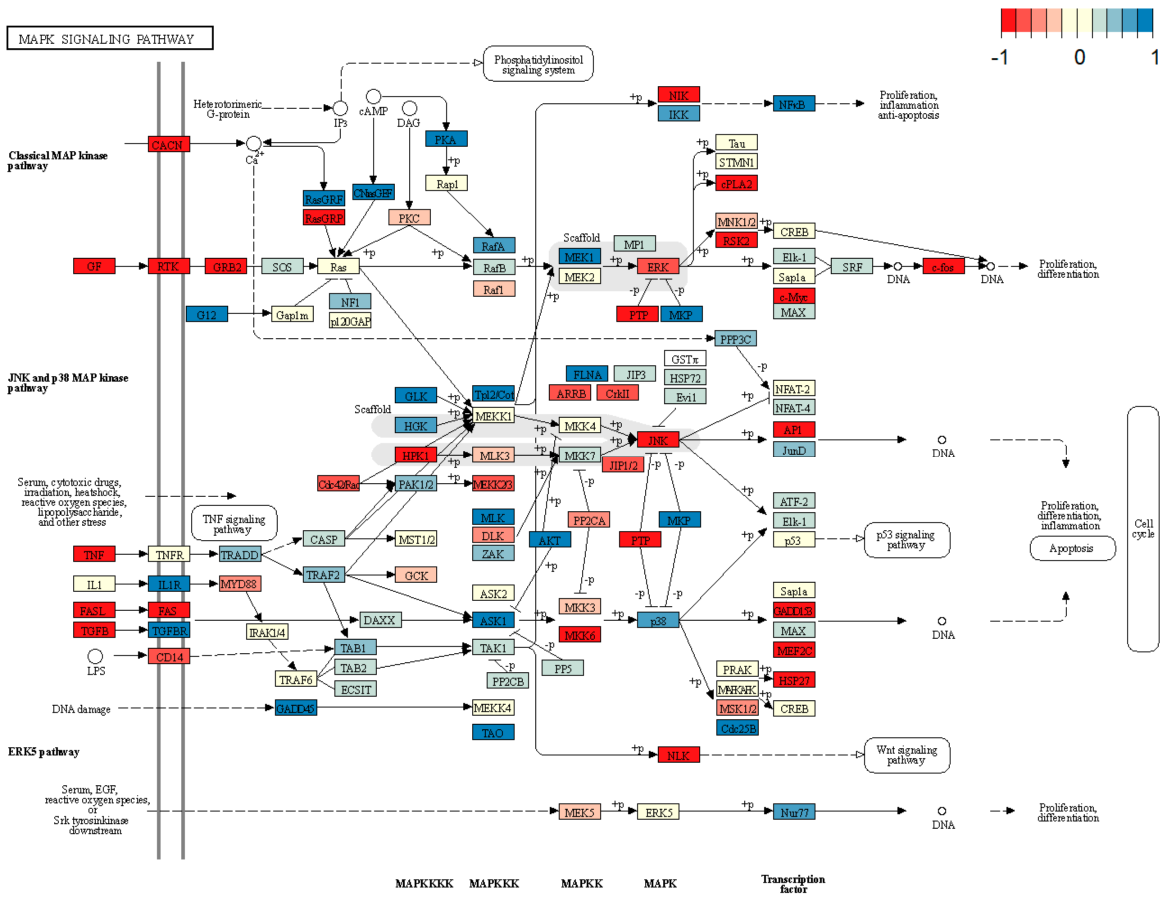

Supplementary Figure S7. MAPK signaling pathway changes between GIST-R1 cells (red, scaled expression < 0) and GIST-R2 cells (blue, scaled expression > 0).

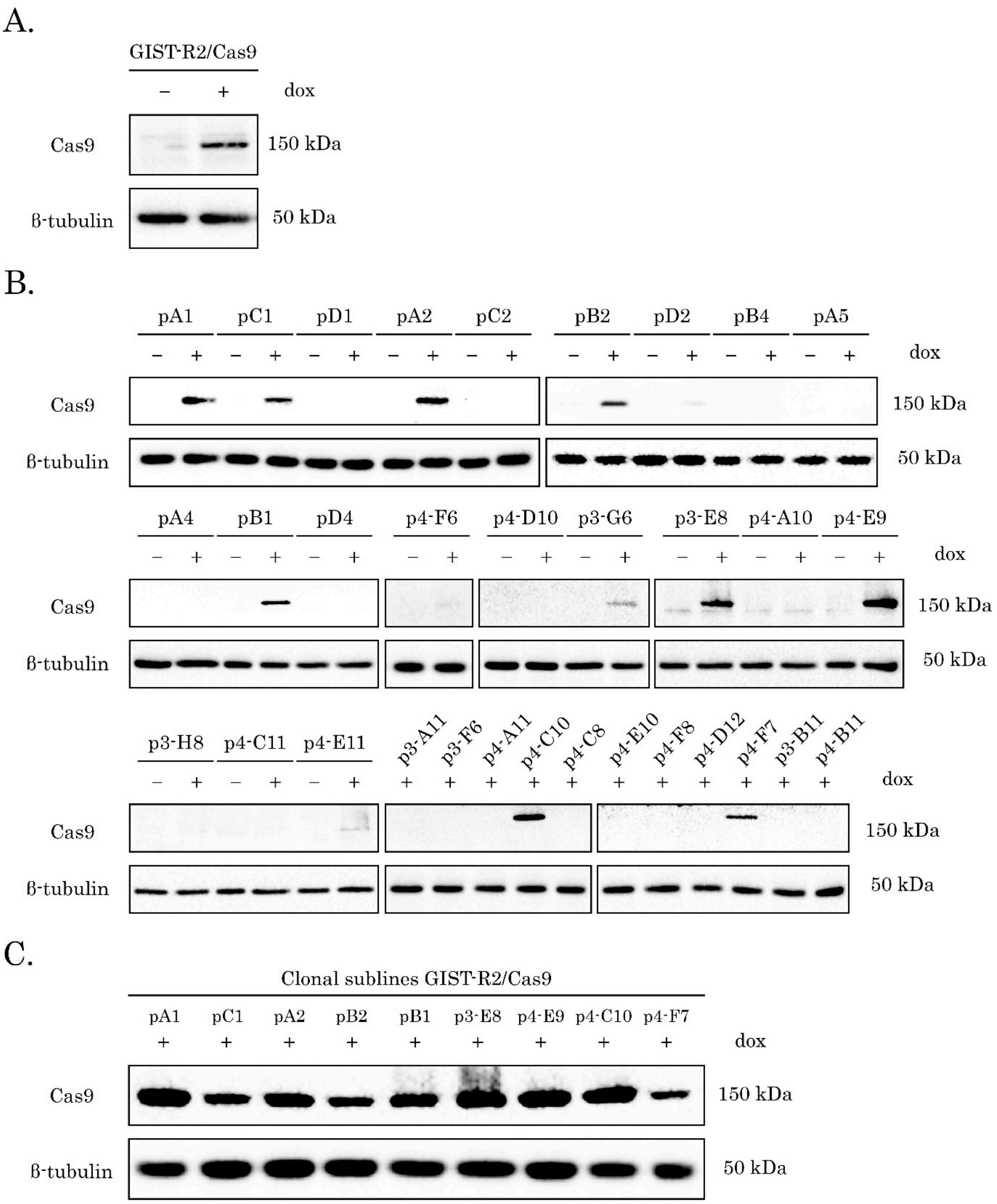

Supplementary Figure S8. Development of GIST-R2 cells expressing doxycycline-dependent endonuclease Cas9. **(A)** Expression of the doxycycline-dependent endonuclease Cas9 in GIST-R2/Cas9 cells after lentiviral transduction and puromycin selection. Cas9 expression was induced in the presence of 1 μg/ml doxycycline for 6 days. **(B)** Expression of the doxycycline-dependent endonuclease Cas9 in GIST-R2/Cas9 clonal sublines. Cas9 expression induction was induced in the presence of 1 μg/ml doxycycline as indicated before. **(C)** Comparative analysis of expression of the doxycycline-dependent endonuclease Cas9 in GIST-R2/Cas9 clonal sublines pA1, pC1 pA2, pB2, pB1, p3-E8, p4-E9, p4-C10 and p4-F7. Induction of Cas9 expression was performed as indicated before. Dox – doxycycline. β-tubulin was amplified as an internal control.

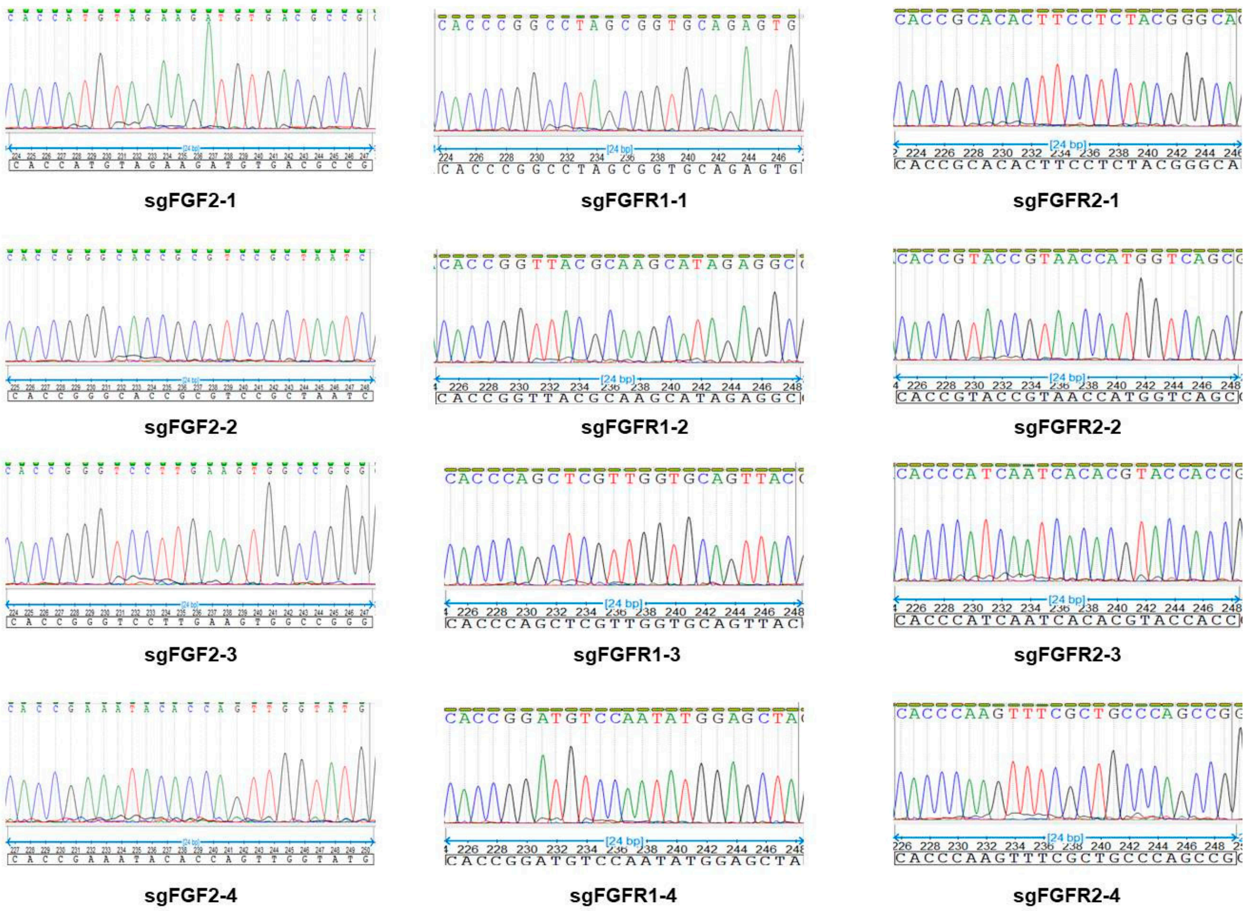

Supplementary Figure S9. Results of Sanger sequencing proving the inserts, which encode sgRNA against *FGF2*, *FGFR1* and *FGFR2* genes (Supplementary Table S1). Data processing was performed in Unipro UGENE 42.0 program software [26-28].

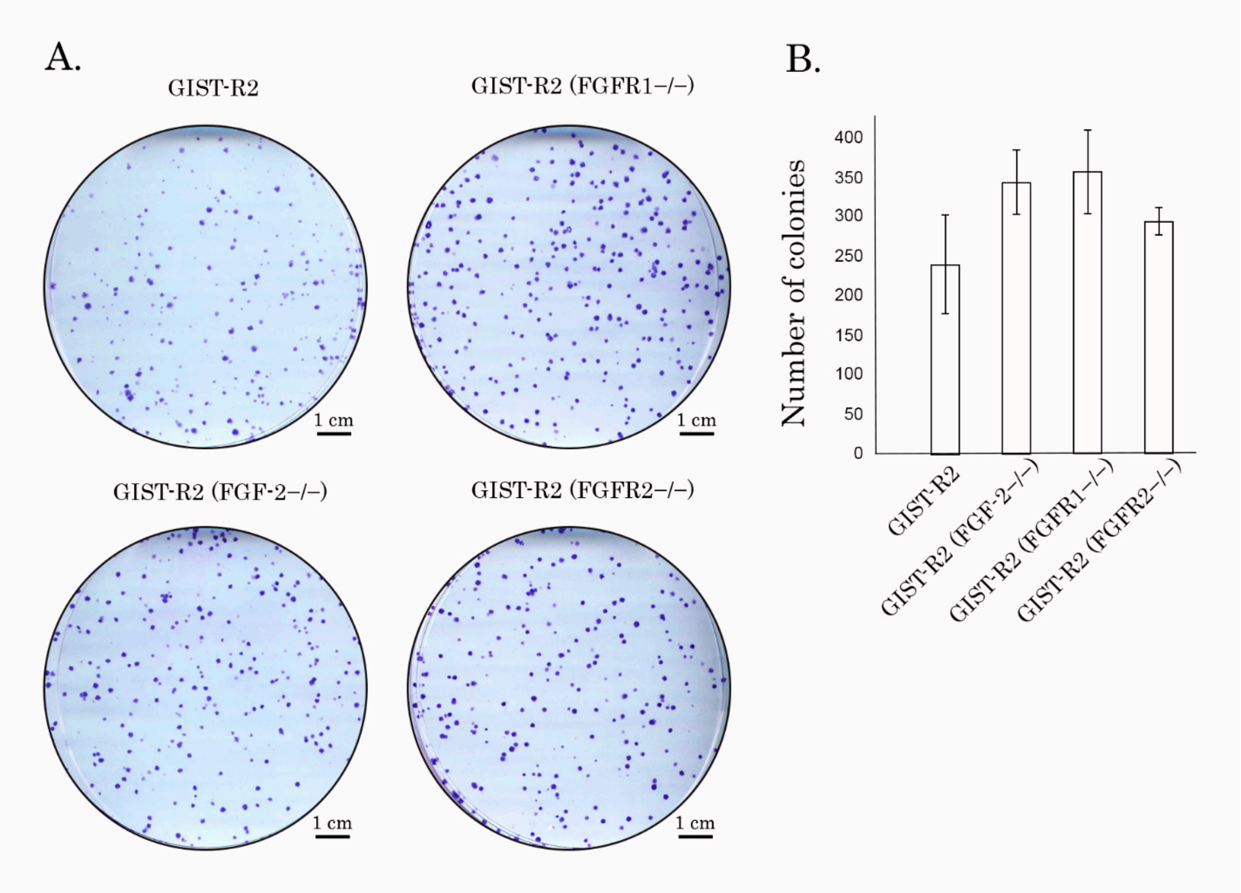

Supplementary Figure S10. Ability of GIST-R2 cells and knockout cells lines (FGF-2−/−, FGFR1−/−, FGFR2−/−) to form the colonies. **(A)** Representative images of the colony formation assay of GIST-R2 cells and knockout cells lines (FGF-2−/−, FGFR1−/−, FGFR2−/−). **(B)** Quantification of the colonies in GIST-R2 cells and knockout cells lines (FGF-2−/−, FGFR1−/−, FGFR2−/−). Data are presented as a median ± SD (n=4).

Supplementary Table S1. Nucleotide sequences of sgRNA oligos for the development of the Cas9-mediated knockout of FGF-2, FGFR1, FGFR2 genes

Target	sgRNA name	Sequences of the forward (F) and reverse (R) sgRNA oligos CACC, AAAC – nucleotides for cloning	Source
FGF-2	F2-1	F: 5'–CACCATGTAGAAGATGTGACGCCG–3' R: 5'–AAACCGGCGTCACATCTTCTACAT–3'	Thermo Fisher Scientific ID CRISPR747906_SGM
	F2-2	F: 5'–CACCGGGCACCGCGTCCGCTAATC–3' R: 5'–AAACGATTAGCGGACGCGGTGCCC–3'	Genscript ID: FGF2 crRNA 1
	F2-3	F: 5'–CACCGGGTCCTTGAAGTGGCCGGG–3' R: 5'–AAACCCCGGCCACTTCAAGGACCC–3'	[19]
	F2-4	F: 5'–CACCGAAATACACCAGTTGGTATG–3' R: 5'–AAACCATACCAACTGGTGTATTTC–3'	Genscript ID: FGF2 crRNA 5
FGFR1	R1-1	F: 5'–CACCCGGCCTAGCGGTGCAGAGTG–3' R: 5'–AAACGCCTCTATGCTTGCGTAACC–3'	[21]
	R1-2	F: 5'–CACCCAGCTCGTTGGTGCAGTTAC–3' R: 5'–AAACGTAACTGCACCAACGAGCTG–3'	Thermo Fisher Scientific ID CRISPR767816_SGM
	R1-3	F: 5'–CACCCAGCTCGTTGGTGCAGTTAC–3' R: 5'–AAACGTAACTGCACCAACGAGCTG–3'	Genscript ID: FGFR1 crRNA 2
	R1-4	F: 5'–CACCGGATGTCCAATATGGAGCTA–3' R: 5'–AAACTAGCTCCATATTGGACATCC–3'	[19]
FGFR2	R2-1	F: 5'–CACCGCACACTTCCTCTACGGGCA–3' R: 5'–AAACTGCCCGTAGAGGAAGTGTGC–3'	Thermo Fisher Scientific ID CRISPR1089779_SGM)
	R2-2	F: 5'–	[22]

		CACCGTACCGTAACCATGGTCAGC–3’<br>R: 5’–<br>AAACGCTGACCATGGTTACGGTAC–3’	
	R2-3	F: 5’–CACCCATCAATCACACGTACCACC–3’<br>R: 5’–<br>AAACGGTGGTACGTGTGATTGATG–3’	Genscript,<br>ID: FGFR2 crRNA 4
	R2-4	F: 5’–<br>CACCCAAGTTTCGCTGCCCAGCCG–3’<br>R: 5’–<br>AAACCGGCTGGGCAGCGAAACTTG–3’	[19]

Supplementary Table S2. Knockout of FGFR2 increases IC50 values of BGJ 398 in GIST-R2 cells.

**Target drug**	**GIST-R2 knockout cell lines**			**GIST-R2**
	**FGF-2−/−**	**FGFR1−/−**	**FGFR2−/−**	
BGJ 398	< 0.0006 μM	1.21 μM ± 0.23	1.93 μM ± 0.36	< 0.0003 μM
Fold increase	2.1	5041.7	8041.7	
