# Supplementary material for: Unraveling the Mechanisms of Sensitivity to Anti-FGF Therapies in Imatinib-Resistant Gastrointestinal Stromal Tumors (GIST) Lacking Secondary KIT Mutations
Source: Cancers (Basel). 2023 Nov 9;15(22):5354. doi: 10.3390/cancers15225354 (PMC10670741; doi:10.3390/cancers15225354)

Uncropped file (Original Images for blots)

Figure 2A.

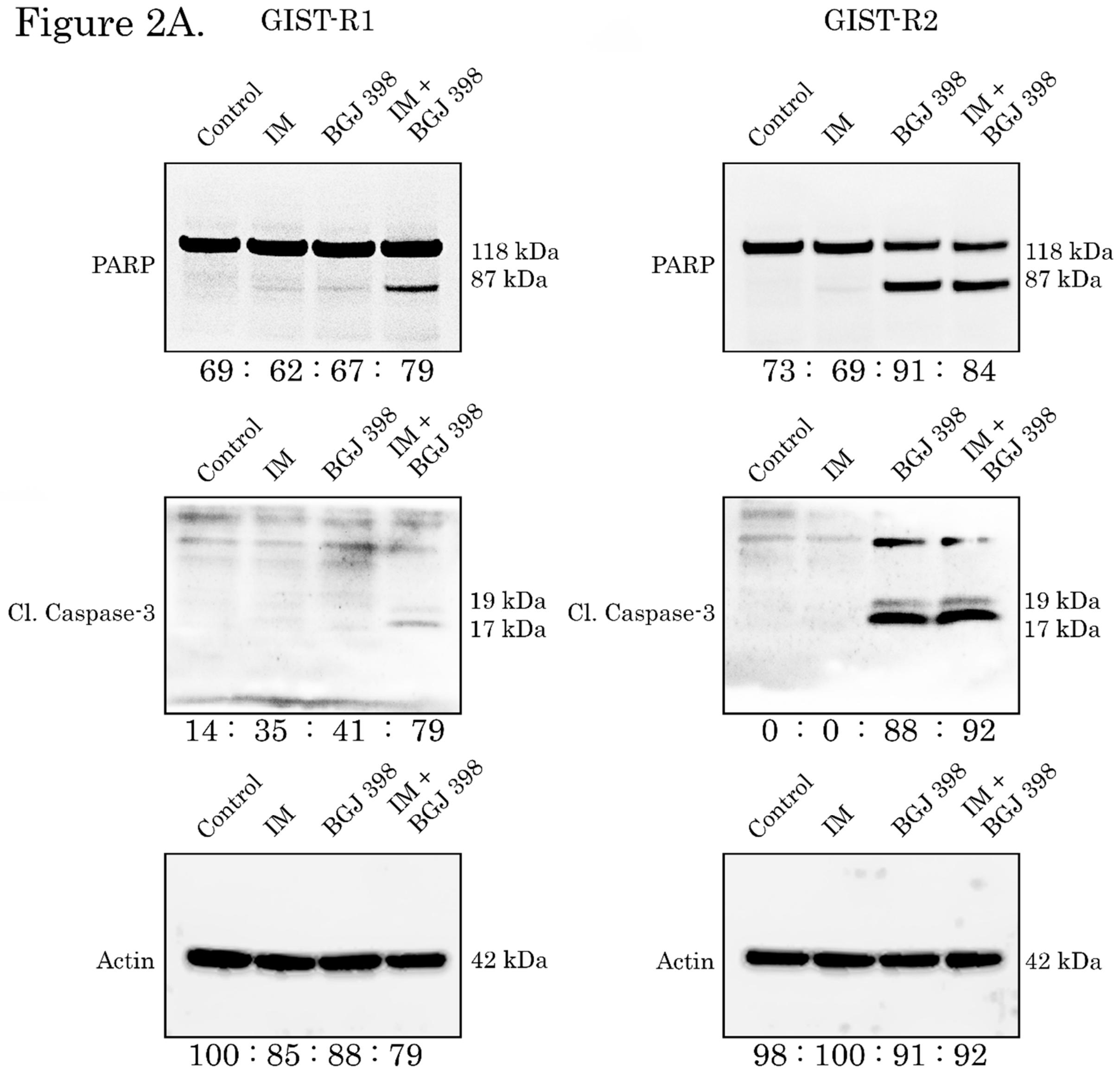

Figure 3 (left).

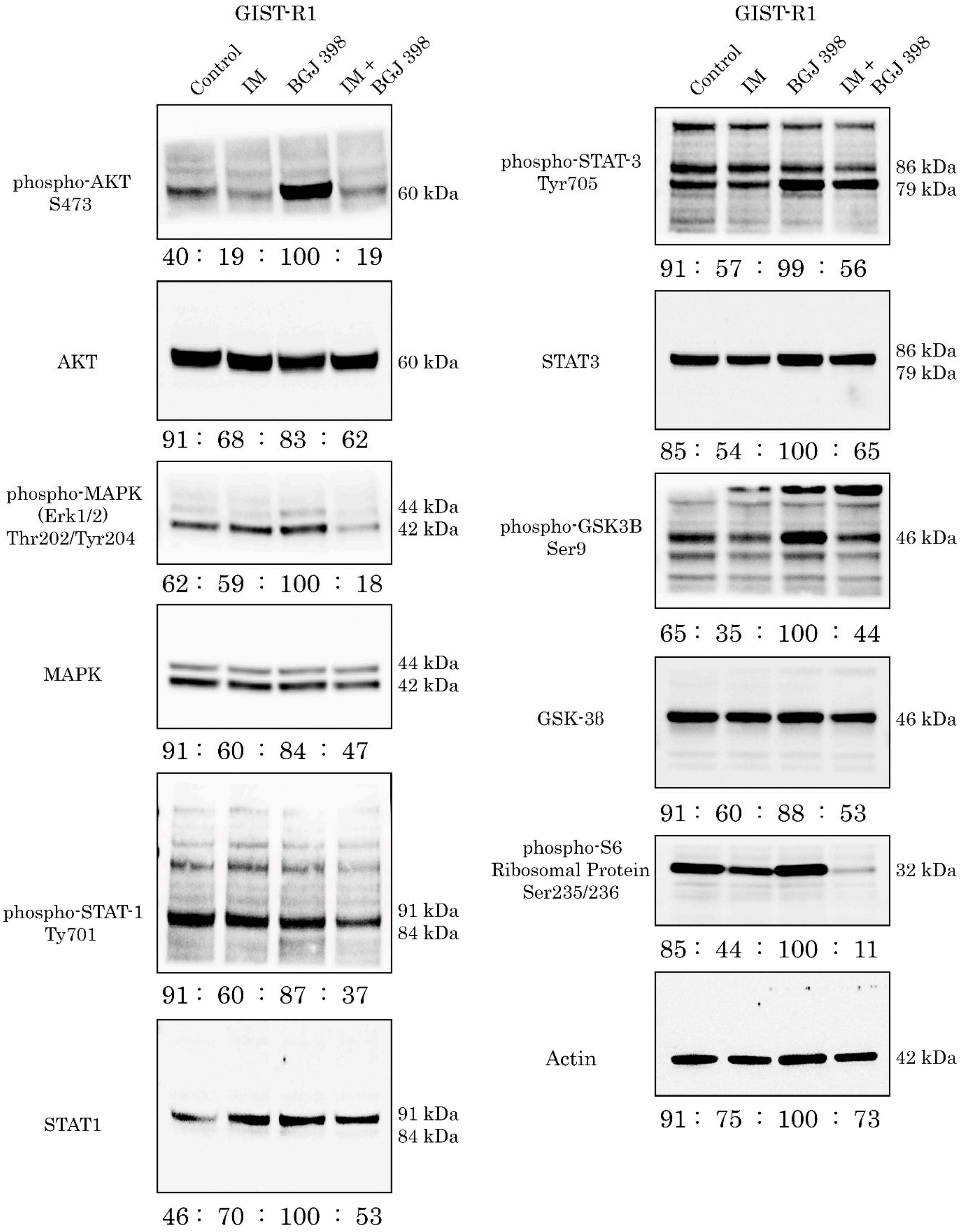

Figure 3 (right).

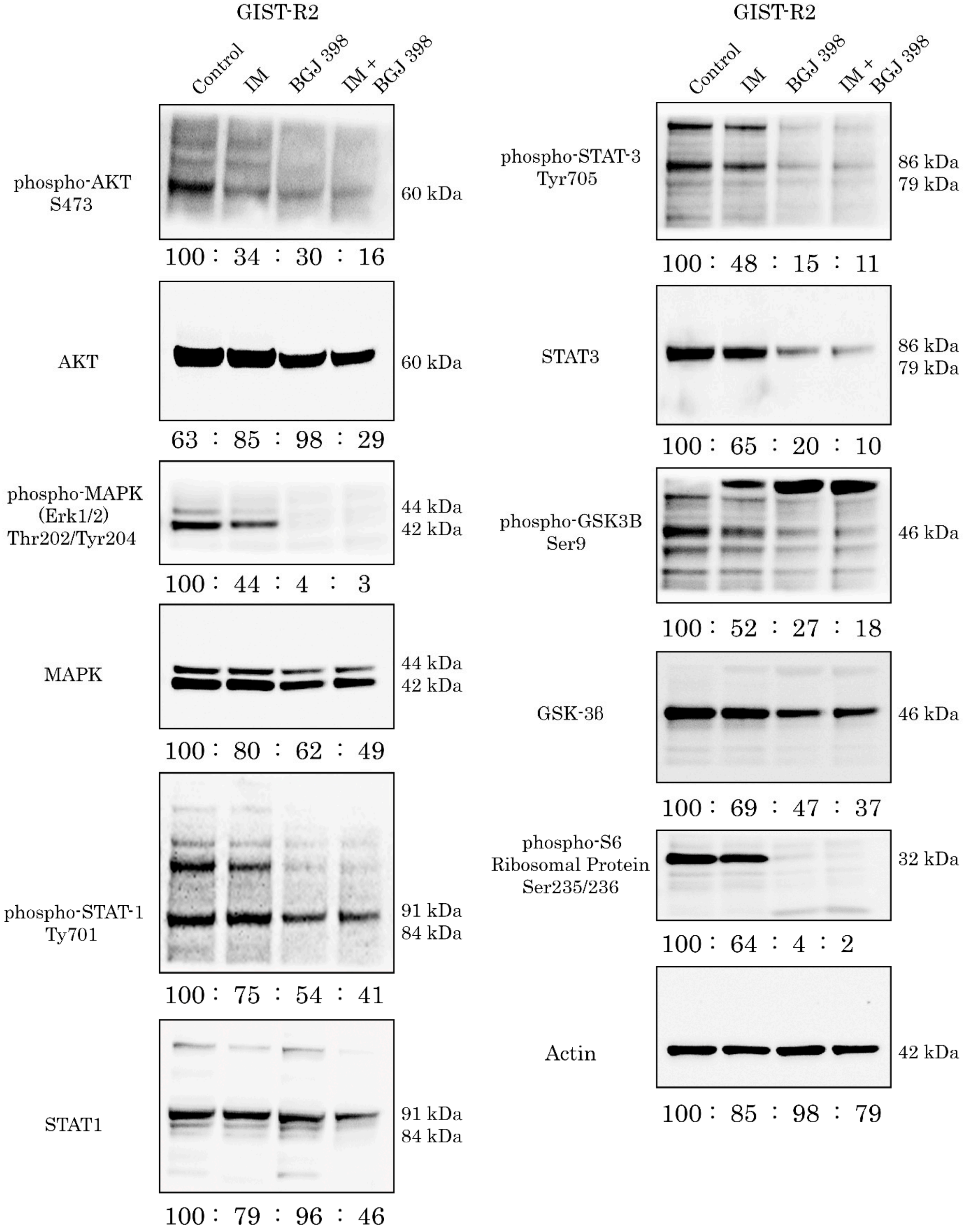

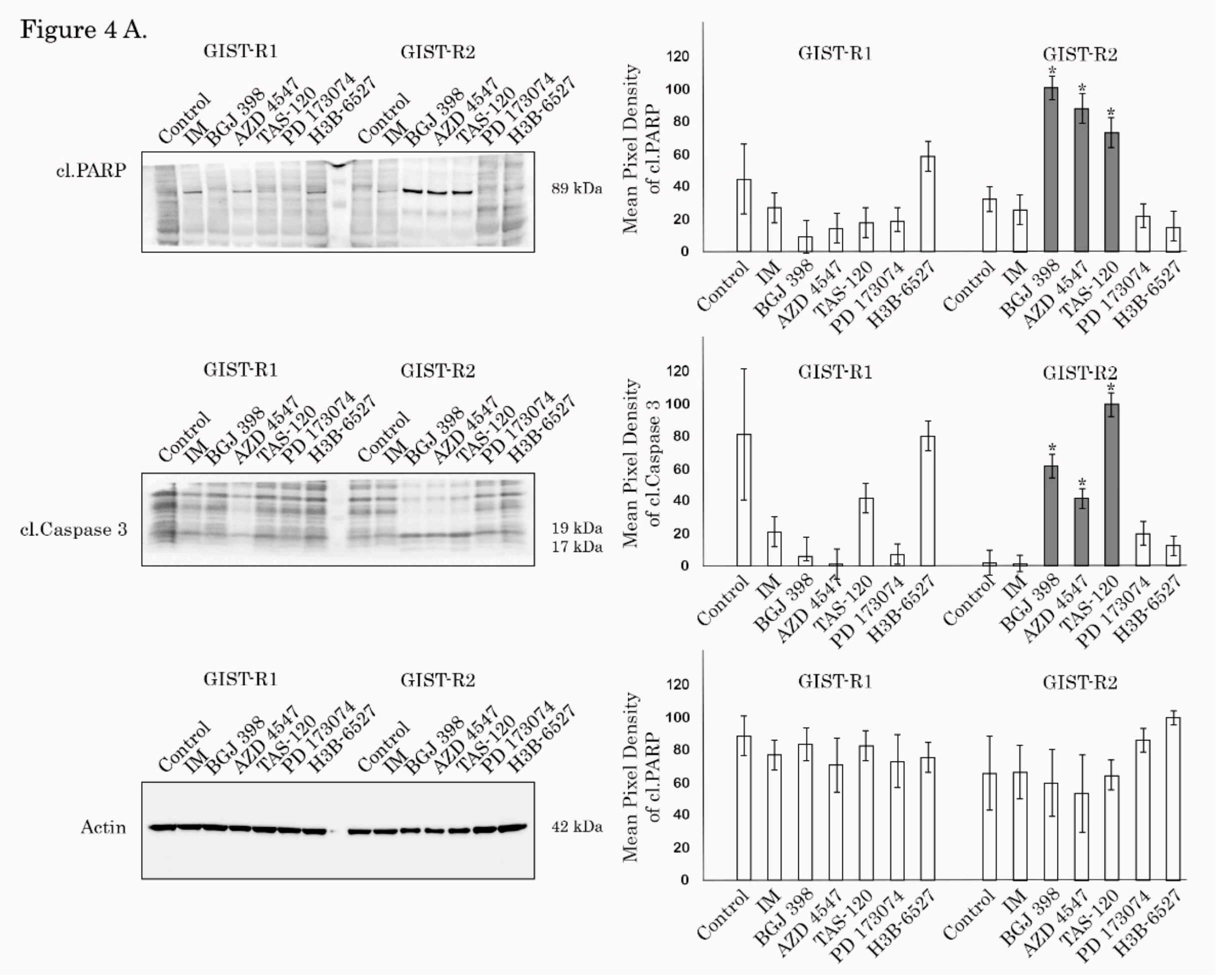

Figure 4 A.
GIST-R1
GIST-R2
Control
IM
BGJ 398
AZD 4547
TAS-120
PD 173074
H3B-6527
cl.PARP
89 kDa
cl.Caspase 3
19 kDa
17 kDa
Actin
42 kDa
Mean Pixel Density of cl.PARP
Mean Pixel Density of cl.Caspase 3

Figure 8A.

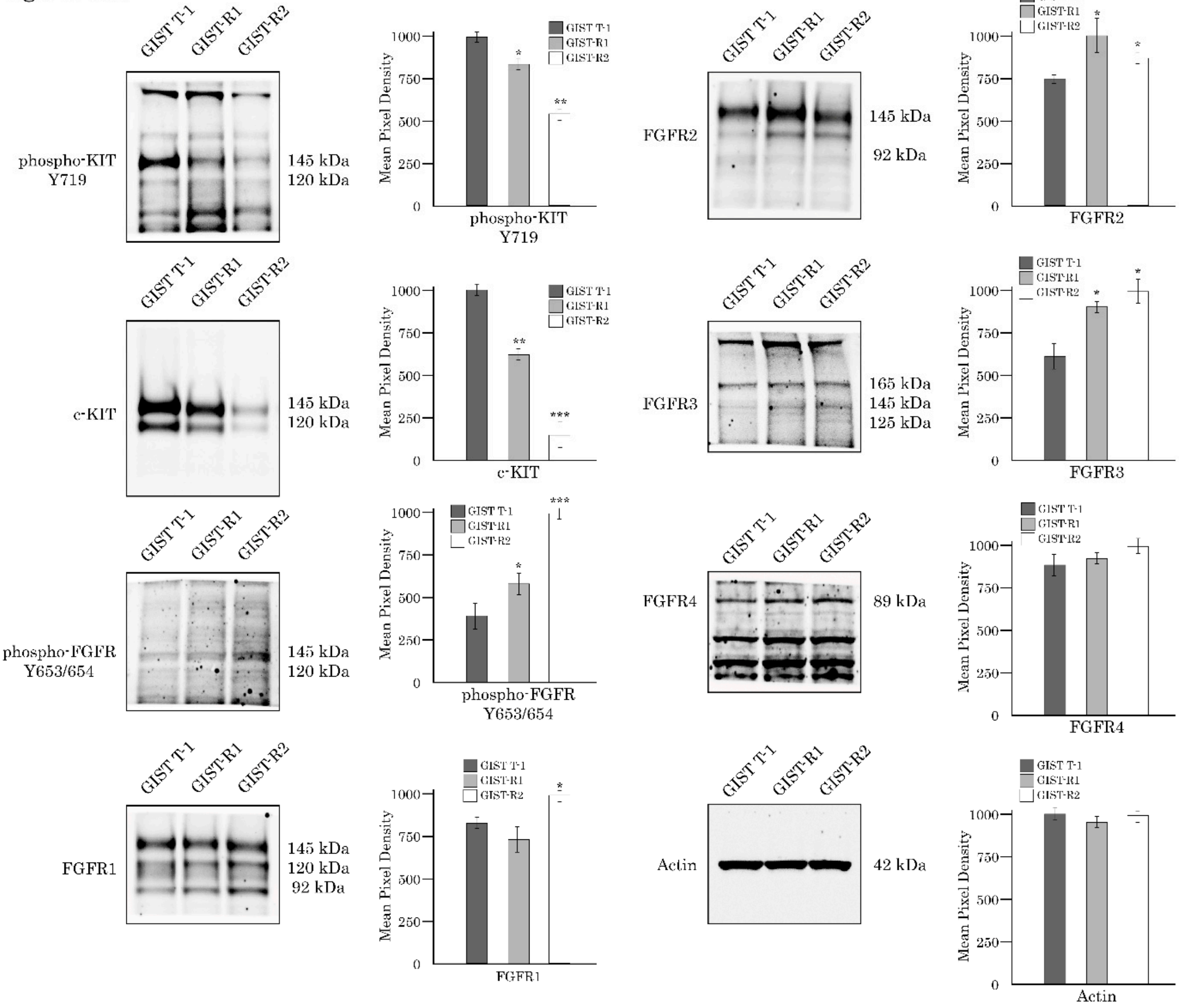

GIST T-1
GIST-R1
GIST-R2
phospho-KIT Y719
145 kDa
120 kDa
c-KIT
phospho-FGFR Y653/654
FGFR1
92 kDa
FGFR2
FGFR3
165 kDa
125 kDa
FGFR4
89 kDa
Actin
42 kDa
Mean Pixel Density
0
250
500
750
1000
*
**
***

Figure 8B.

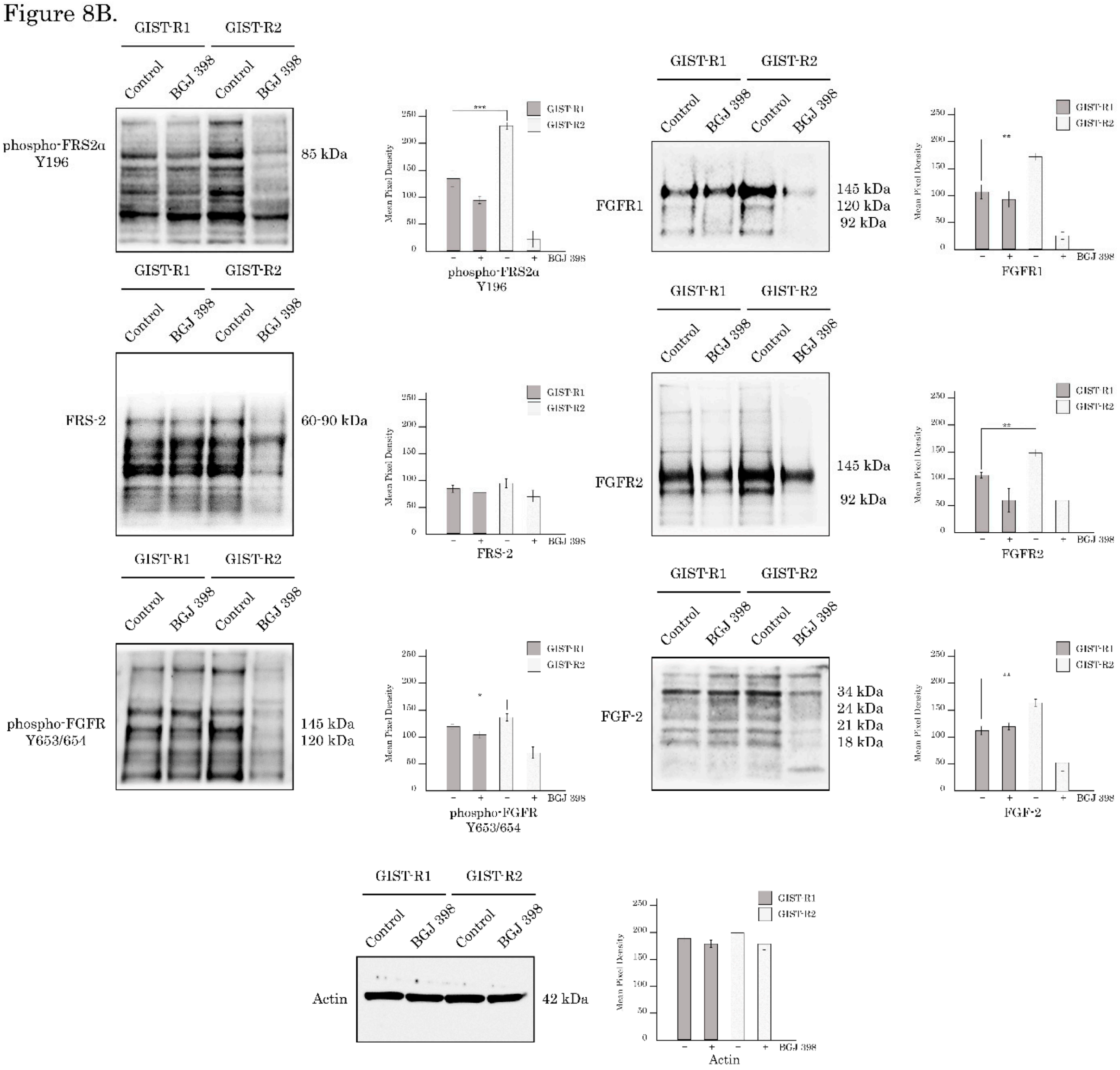
GIST-R1
GIST-R2
Control
BGJ 398
phospho-FRS2α Y196
85 kDa
FRS-2
60-90 kDa
phospho-FGFR Y653/654
145 kDa
120 kDa
FGFR1
145 kDa
120 kDa
92 kDa
FGFR2
145 kDa
92 kDa
FGF-2
34 kDa
24 kDa
21 kDa
18 kDa
Actin
42 kDa
Mean Pixel Density
BGJ 398
FGFR1
FGFR2
FGF-2

Figure 11A.

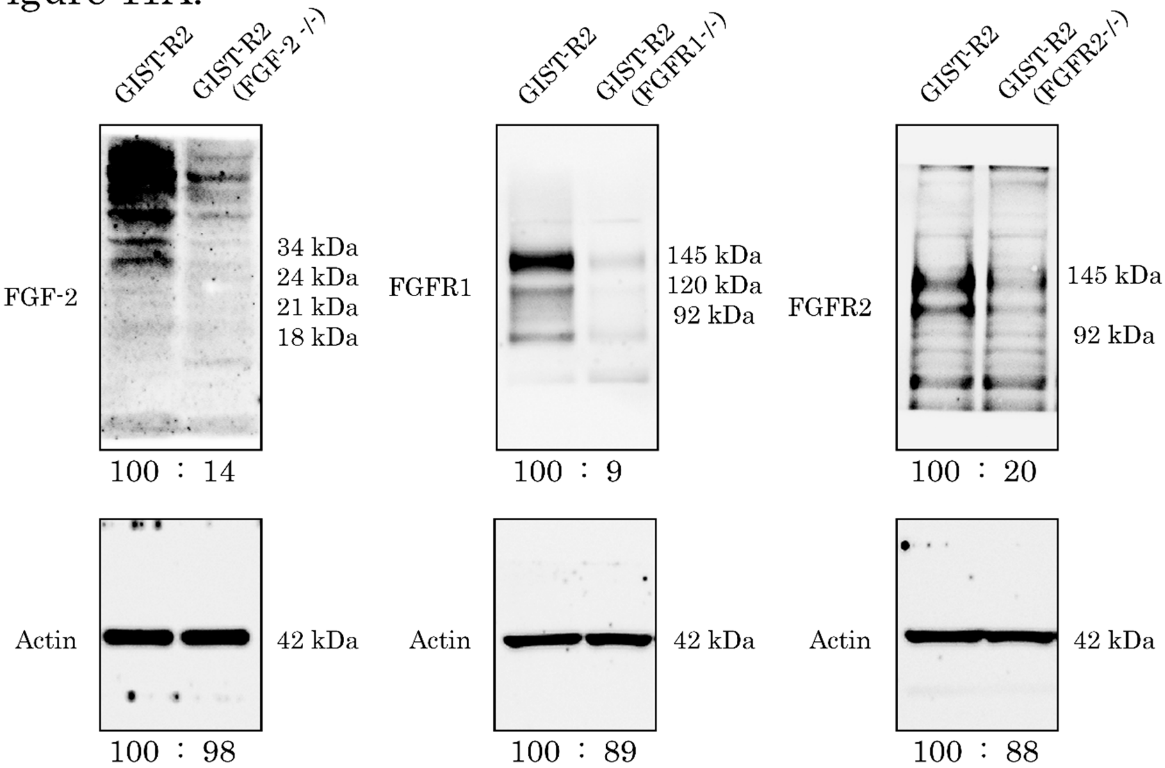
GIST-R2
GIST-R2 (FGF-2 -/-)
FGF-2
34 kDa
24 kDa
21 kDa
18 kDa
100 : 14
Actin
42 kDa
100 : 98
GIST-R2
GIST-R2 (FGFR1-/-)
FGFR1
145 kDa
120 kDa
92 kDa
100 : 9
Actin
42 kDa
100 : 89
GIST-R2
GIST-R2 (FGFR2-/-)
FGFR2
145 kDa
92 kDa
100 : 20
Actin
42 kDa
100 : 88

Figure 12C.

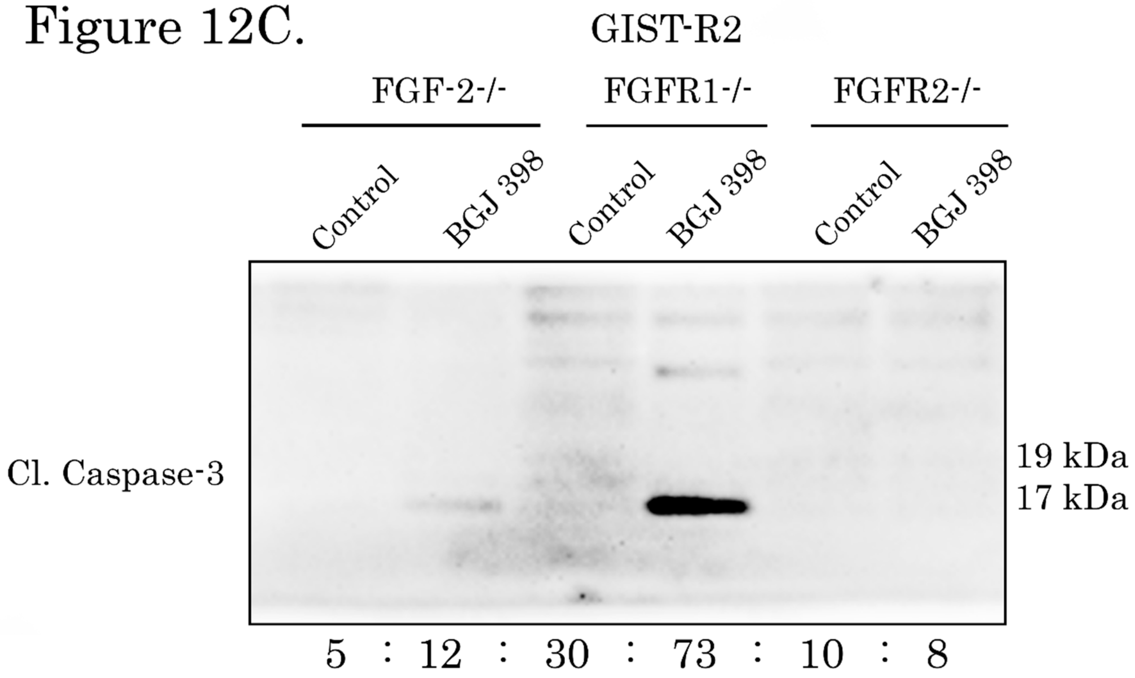
GIST-R2
FGF-2-/-
FGFR1-/-
FGFR2-/-
Control
BGJ 398
Control
BGJ 398
Control
BGJ 398
Cl. Caspase-3
19 kDa
17 kDa
5 : 12 : 30 : 73 : 10 : 8

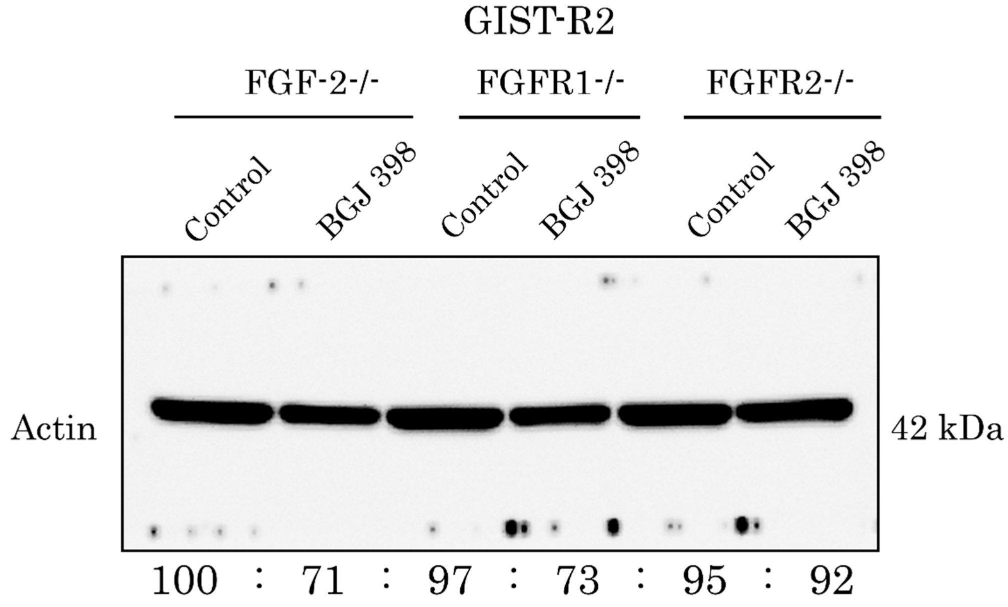
GIST-R2
FGF-2-/-
FGFR1-/-
FGFR2-/-
Control
BGJ 398
Control
BGJ 398
Control
BGJ 398
Actin
42 kDa
100 : 71 : 97 : 73 : 95 : 92

Supplementary figure 1B.

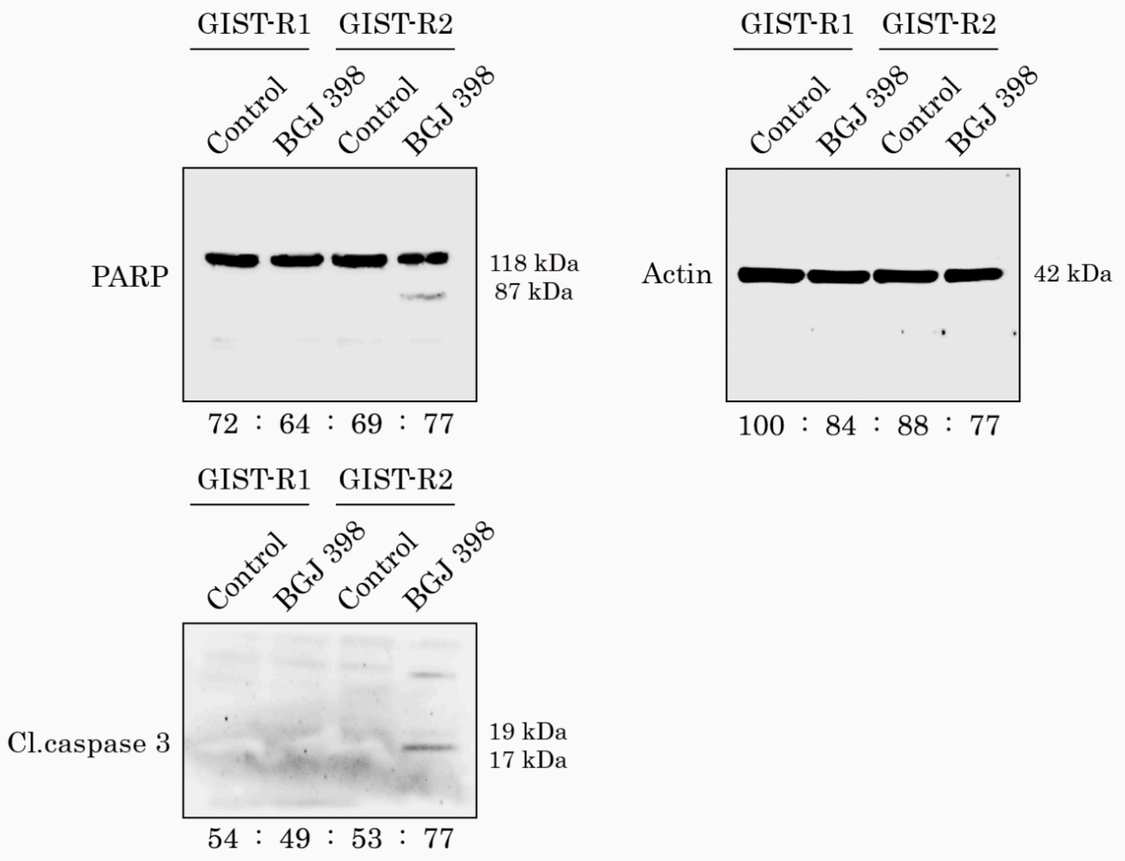

Supplementary figure 8A.

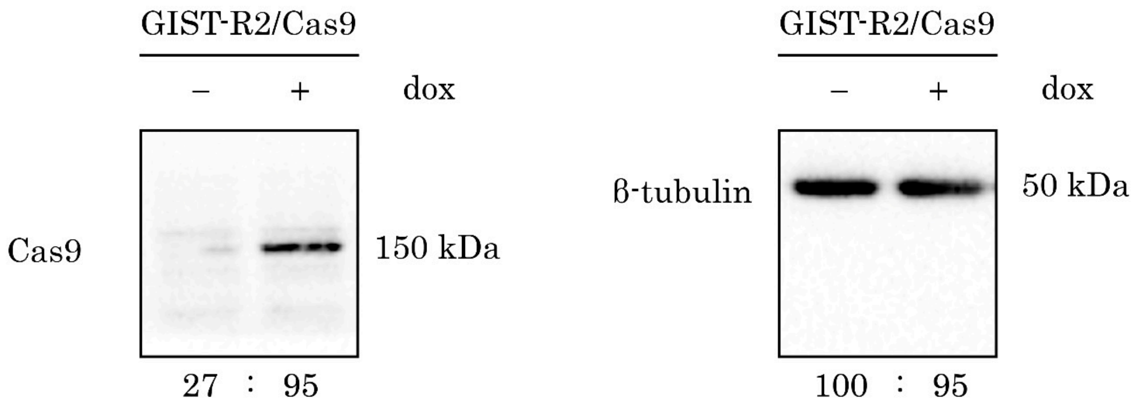

Supplementary figure 8B.

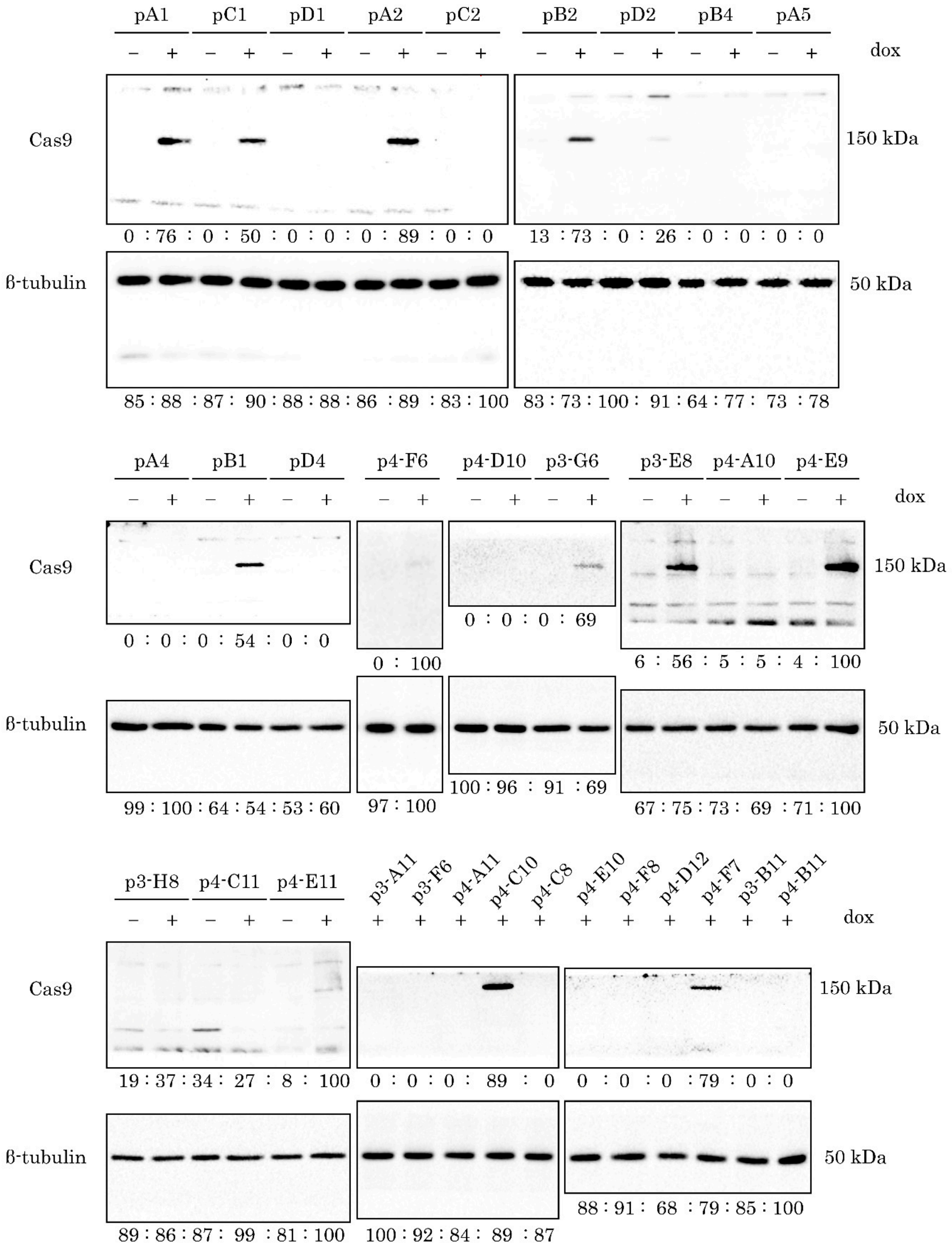

Supplementary figure 8C.

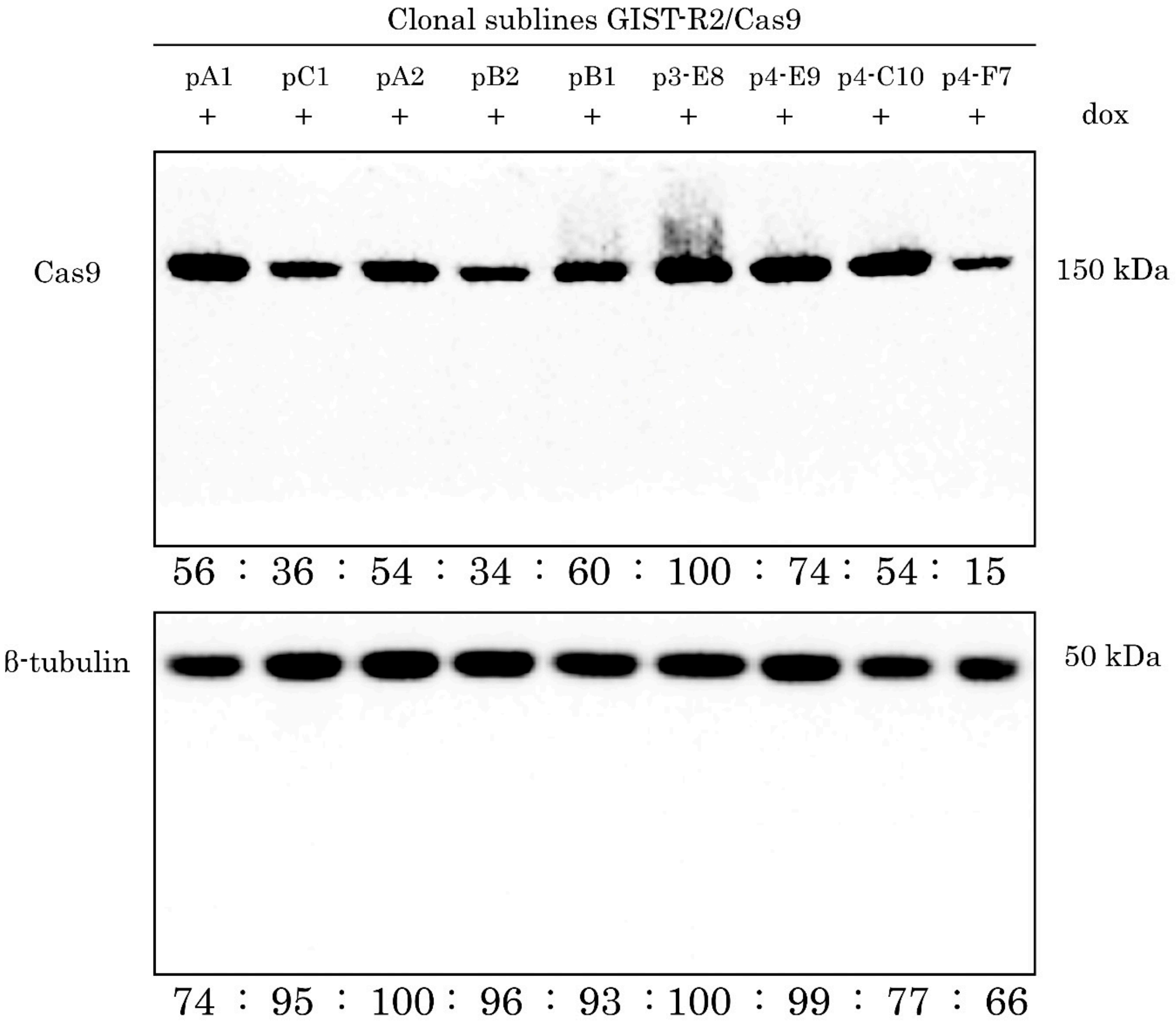

Supplement: Supplementary file 1 [file cancers-15-05354-s001.zip › File S2.pdf]
